# Tissue Microarray-Based Digital Spatial Profiling of Benign Breast Lobules and Breast Cancers: Feasibility, Biological Coherence, and Cross-Platform Benchmarks

**DOI:** 10.3390/cancers17233797

**Published:** 2025-11-27

**Authors:** Mark E. Sherman, Jodi C. Carter, Robert A. Vierkant, Melody Stallings-Mann, Laura Pacheco-Spann, Stacey J. Winham, Celine M. Vachon, Chen Wang, Matthew R. Jensen, Melissa A. Troester, Amy C. Degnim, E. Aubrey Thompson, Jennifer Kachergus, Ji Shi, Derek C. Radisky

**Affiliations:** 1 Quantitative Health Sciences, Mayo Clinic Alix School of Medicine and Health Sciences, Jacksonville, FL 32225, USApachecospann.laura@mayo.edu (L.P.-S.); 2 Department of Laboratory Medicine and Pathology, University of Alberta, 114 Street and 89 Avenue, Edmonton, AB T6G 2M7, Canada; 3 Quantitative Health Sciences, Mayo Clinic Alix School of Medicine and Health Sciences, Rochester, MN 55905, USA; 4 Cancer Biology, Mayo Clinic, Jacksonville, FL 32225, USA; 5 Division of Epidemiology, Mayo Clinic Alix School of Medicine and Health Sciences, Rochester, MN 55905, USA; 6 Lineberger Comprehensive Cancer Center, University of North Carolina at Chapel Hill, Chapel Hill, NC 27599, USA; 7 Department of Pathology and Laboratory Medicine, University of North Carolina at Chapel Hill, Chapel Hill, NC 27599, USA; 8 Department of Surgery, Mayo Clinic Alix School of Medicine and Health Sciences, Rochester, MN 55905, USA

**Keywords:** digital spatial profiling, tissue microarray, terminal duct lobular unit, benign breast disease, breast cancer, immunohistochemistry, multiplex immunofluorescence (OPAL), reproducibility, cross-platform agreement, biomarker discovery

## Abstract

Background: Discovering risk biomarkers in small benign breast disease (BBD) biopsies is constrained by scarce tissue and microanatomic heterogeneity of terminal duct lobular units (TDLUs). We tested whether tissue-sparing tissue microarray (TMA)–based Digital Spatial Profiling (DSP) can deliver reproducible, biologically coherent protein measurements across benign lobules and breast cancers (BCs), and how well DSP aligns with standard immunoassays. Methods: We performed a pilot using tissues from the Mayo Clinic BBD cohort using TMAs representing four contexts: terminal duct lobular units (TDLUs) from BBD biopsies preceding BC and matched BBD-controls, subsequent BCs, and BC-associated TDLUs. We profiled 79 proteins by DSP (37 retained after QC) and benchmarked against chromogenic IHC and OPAL immunofluorescence. Reproducibility was evaluated using intraclass correlation coefficients (ICCs), cross-platform agreement (weighted kappa), marker correlations, and mixed-effects models with false-discovery-rate (FDR) control. Results: We analyzed 368 BBD-TDLU cores (88 cases; 88 controls), 204 BC cores and 110 BC-associated TDLU cores. ICCs were highest in BC tissues, and lower in BC-associated TDLUs and BBD-TDLUs. Agreement was slight–to-fair in TDLUs but moderate (ER/PR) to substantial (BCL2) in BC. DSP recapitulated expected immunologic correlations (CD45 with T-cell, B-cell, and macrophage markers) and tissue-type gradients (BC > BC-associated TDLUs > BBD-TDLUs). Exploratory case–control differences in BBD-TDLUs did not persist after FDR control. Conclusions: TMA-based DSP is feasible in archival breast tissues and yields biologically coherent, cross-platform-benchmarked profiles that are particularly robust in BC, while conserving scarce TDLUS and clarifying current limits of single-marker risk stratification from benign lobules. These data provide a foundation for refined sampling and expanded panels in future TDLU-focused studies.

## Simple Summary

Most breast biopsies show non-cancerous (benign) changes, yet some women later develop breast cancer. Predicting who is at higher risk from these small tissue samples is difficult because the relevant structures are tiny and easily exhausted by testing. We evaluated whether a multiplex technology called digital spatial profiling can measure many proteins at once on tissue microarrays built from benign breast lobules, nearby tissue next to cancers, and cancers themselves. We also compared the results to standard lab tests. The approach worked well overall and reproduced known biology, especially in cancer tissue. Signals in benign lobules were more variable and did not, on their own, distinguish women who later developed cancer from matched controls in this pilot. These findings show that high-plex, tissue-conserving profiling is feasible and biologically coherent, and they outline concrete ways to improve sampling and marker panels for future risk-prediction studies.

## 1. Introduction

Benign breast disease (BBD) encompasses lesions with variable associations with subsequent breast cancer (BC) [1,2,3,4]; accurate risk stratification can guide surveillance, prevention, and reassurance [5,6]. Discovery of biomarkers in BBD biopsies that predict breast cancer (BC) risk or inform carcinogenic mechanisms could guide precision management and prevention. Most BBD biopsies sample both the lesion and the surrounding, normal appearing terminal duct lobular units (TDLUs). Because TDLUs comprise the bulk of epithelium throughout the breast and are the microanatomical source of most BC precursors, analysis of these structures could have value in risk prediction and define different pathways of carcinogenesis. However, research on this topic has been constrained: (1) availability of benign tissues with annotated clinical follow-up is limited; (2) small structures of interest are exhausted by repeated histologic sectioning for single-plex assays; (3) bioinformatic deconvolution to account for cellular heterogeneity in bulk sequencing data is imprecise [7,8,9]; and (4) microdissection and DNA/RNA sequencing in large studies is impractical [10,11].

TDLUs are comprised of acini and ducts lined by luminal and myoepithelial cells within an immune-rich stroma. Starting in the peri-menopausal period, TDLUs undergo physiological age-related involution, characterized by a reduction in acini content and size, and ultimately, replacement by fibrofatty stroma [12]. Delayed age-related involution has been associated with elevated BC risk [13,14] and with circulating hormones and growth factors linked to increased BC risk [4,15,16,17,18], suggesting that delayed age-related involution may represent an intermediate marker of carcinogenesis [19]. In clinical BBD biopsies, increased epithelial proliferation [16], estrogen receptor (ER) expression [15] and insulin-like growth factor receptor (IGF1R) [20] in luminal epithelial cells of TDLUs have been associated with subsequent risk, and immune-cell composition in benign lobules may carry risk information [21]. Finally, four independent studies have demonstrated reduced levels of TDLU involution in benign tissues surrounding triple-negative as compared with luminal BCs [22,23], consistent with proposed differences in the etiology and pathogenesis of these BC subtypes [22,24,25,26,27,28].

Analysis of biomarkers in BBD biopsies is challenging. Although immunohistochemistry (IHC) enables assessment of protein expression in individual cells, quantitation of chromogenic signals has limited sensitivity, precision, and linearity, and evaluating whole sections for single IHC biomarkers is time-consuming, effortful and costly [29]. We propose that high-plex biomarker approaches combined with tissue microarrays (TMAs) offer a potential path forward [30,31,32,33,34]. The NanoString Digital Spatial Profiling (DSP) enables multiplex protein quantification on formalin-fixed sections while conserving tissue and preserving context [35,36,37,38,39,40,41,42]. Accordingly, within the well-characterized Mayo BBD cohort [43], we tested whether constructing TDLU-rich TMAs and applying automated, high-plex DSP, benchmarked against chromogenic immunohistochemistry (IHC) and OPAL immunofluorescence staining, can address key barriers to biomarker discovery in small archival samples. We proposed that multiplex assays on TMAs would conserve scarce microanatomic structures and enable high-throughput, quantitative immunofluorescent readouts with improved dynamic range relative to single-plex IHC. We sampled four tissue contexts: (1) TDLUs within BBD biopsies that preceded BC (BBD-cases); (2) TDLUs within a matched set of BBD biopsies from BC-free women (BBD-controls); (3) BCs diagnosed after the BBD-case biopsies; and (4) TDLUs surrounding BCs from BBD-cases, referred to herein as “BC-associated TDLUs”. In this study, ‘spatial profiling’ refers primarily to quantitative, high-plex protein measurements obtained from anatomically defined microenvironments—benign TDLUs, tumor-adjacent TDLUs, and cancers—rather than whole section averages. By combining TMAs with GeoMx DSP, we targeted ROIs over specific microanatomical structures (lobules vs. invasive tumor nests) and tissue contexts (benign vs. tumor adjacent vs. malignant), allowing us to compare protein expression patterns and immune relationships across these distinct spatial compartments while conserving tissue. Our objectives were to: (i) assess the feasibility and reproducibility of DSP in TDLUs and BC cores, (ii) quantify cross-platform agreement between DSP and chromogenic IHC/OPAL immunofluorescence, and (iii) explore whether TDLU protein profiles in BBD biopsies differentiate cases from matched controls. While not designed for risk prediction, this technical pilot is intended to inform assay, sampling, and analysis choices for larger studies aimed at developing TDLU-based biomarkers to support clinical risk stratification in BBD.

## 2. Materials and Methods

### 2.1. Study Cohort and Design

We conducted a nested, frequency-matched case–control study within the Mayo Clinic Benign Breast Disease (BBD) cohort (diagnostic years 1994–2001) [43]. We initially identified 100 women whose BBD biopsy preceded a subsequent breast cancer (BC; “BBD-cases”) and 100 women who remained cancer-free (“BBD-controls”), frequency-matched on calendar period (1994–1997 vs. 1998–2001), age (<50 vs. ≥50 years), BBD severity (non-atypical BBD vs. atypical hyperplasia), and length of follow-up. Exclusions for missing risk-factor questionnaires or <10 years of follow-up yielded a final analytic set of 88 cases and 88 controls. Among cases, 52 (59%) had archival FFPE material available from the subsequent cancer surgery to sample BC and BC-associated TDLUs. Hematoxylin-and-eosin sections from BBD biopsies, BCs, and BC-associated TDLUs were reviewed to select TDLU-rich regions for tissue microarray (TMA) construction; reviewers were masked to clinical outcomes at the time of selection. Study procedures were approved by the Mayo Clinic Institutional Review Board.

### 2.2. Construction of Tissue Microarrays (TMAs)

For all BBD biopsies, invasive cancers, and cancer surgeries, diagnostic H&E slides were reviewed by M.E.S. and J.C.C., masked to clinical outcomes, to identify TDLU-rich benign regions, invasive cancer regions, and morphologically benign lobules in the breast tissue surrounding cancers. From each eligible specimen, we sought to sample three tissue contexts: (i) TDLUs from BBD biopsies (all participants), (ii) BC tissues (cases only), and (iii) BC-associated TDLUs located approximately 1.0 mm and 2.0 mm from the tumor edge (cases only). When tissue permitted, multiple punches per specimen and per context were taken to enable within-patient reproducibility estimates. TMAs were constructed using an ISE Galileo CK4500 (Integrated Systems Engineering, Philadelphia, PA, USA) with a 1.0-mm diameter needle in grids up to 11 rows × 17 columns. Five BBD TMAs and five BC/BC-associated TDLU TMAs were created (1–4 cores per parent block). Tonsil, cervix, and placenta tissues were included as technical controls; liver tissue aided orientation.

### 2.3. NanoString Digital Spatial Profiling (DSP)

TMA sections used for DSP were stained sections with an antibody cocktail of visualization markers, including three fluorophore-labeled antibodies against cytokeratin (epithelial cell marker), CD45 (pan-leukocyte marker), and CD68 (macrophage marker), plus the UV-compatible nuclear dye SYTO13 (Thermo Fisher Scientific, Waltham, Massachusetts, USA). The sections were also stained with a second cocktail of profiling reagents containing 79 target antibodies, each labeled with a unique oligonucleotide barcode linked to the antibody via a UV-labile cross-linker [36,37,38,44]. The 79 protein panel was assembled to capture (i) clinically established breast cancer markers (e.g., ER, PR, HER2, Ki67, BCL2), (ii) immune and stromal markers spanning major leukocyte lineages and antigen presenting cells (e.g., CD3, CD4, CD8, CD20, CD45, CD11c, CD68, HLA DR), and (iii) additional signaling and checkpoint molecules relevant to breast carcinogenesis and host response (e.g., STING, CTLA4, Tim3, NF1). Panel composition was informed by prior work in benign breast disease and breast cancer, published GeoMx best practice recommendations [44], and vendor-provided validation for FFPE compatibility and detection performance. For each core, a single circular region of interest (ROI; 600 µm diameter) was placed over the TDLU (or BC) area containing the most epithelial cells, as assessed from the morphology markers. For BBD TDLU cores, ROIs were placed over morphologically benign lobules within the BBD biopsy that contained the greatest density of intact acini and ducts and no carcinoma or atypia. For BC-associated TDLU cores, ROIs were centered on benign appearing lobules located approximately 1 mm and 2 mm from the invasive tumor edge, as defined during TMA construction. For BC cores, ROIs were placed within invasive carcinoma, avoiding necrosis, in situ components, and large vessels. In all cases, ROI placement was guided by the cytokeratin, CD45, and CD68 morphology markers to maximize epithelial content while capturing the local immune-rich stroma. Oligo tags released by UV illumination were collected and analyzed using NanoString nCounter technology. The GeoMx DSP platform enables high-plex protein/RNA profiling on FFPE tissue using UV-cleavable oligonucleotide tags and morphology-guided ROI selection [38,45]. Raw ROI-level protein counts were first background-adjusted using the isotype negative control antibodies supplied in the GeoMx protein panel. For each ROI, the three IgG isotype controls (mouse IgG1, mouse IgG2a, rabbit IgG) were used to estimate local background, and target signals were normalized relative to this background before transformation. Background-adjusted expression values were then transformed to the log2 scale and used for all downstream analyses. We selected this negative-control-based normalization because it relies on controls present in every ROI, does not assume that housekeeping proteins are constant across benign lobules and cancers, and preserves a scale that can be compared directly with chromogenic IHC and OPAL immunofluorescence readouts.

DSP quality control (QC): Background correction used the three isotype IgG controls supplied in the protein panel (mouse IgG1, mouse IgG2a, rabbit IgG), consistent with published GeoMx guidance to evaluate and normalize using isotype/negative probes before downstream analyses [37]. We filtered low-expressing targets by comparing each biomarker to three isotype negatives (mouse IgG1, mouse IgG2a, rabbit IgG), computing (i) the mean difference between log-expression and the average of negative controls across all cores and (ii) the proportion of cores with expression higher than all three positive controls. Biomarkers with a mean difference <0 and with <5% of cores above positive controls were excluded. Of 79 assayed proteins, 40 (including housekeeping/positive controls) passed QC; after removing positive controls, 37 non-control proteins were retained for analysis.

### 2.4. Immunohistochemistry (IHC)

FFPE TMA sections (4-um) were deparaffinized, rehydrated, and subjected to heat-mediated antigen retrieval in citrate buffer, pH = 6.0 per manufacturer’s directions (DAKO). Sections were incubated with the following primary antibodies at room temperature for 1 h: ER: Abcam–ab16660; Ki67: Agilent/Dako–M1740; anti-mouse BCL2: Agilent/Dako–IR61461; CD3: Novus–NB600-1441, titered on human tonsil, anti-rabbit; CD45: Agilent/Dako–IR75161; p16: Abcam Ab54210 1:1600; anti-mouse, CD20: IRS60461-2 Dako/Agilent; and PR: Cell signaling technologies, 1:750, 87,575. Stained sections were rinsed in Tris-buffered saline/Triton-X-100 (TBST) and incubated with secondary antibody for 30 min. Following a repeat rinse in TBST, sections were incubated in 3,3′-diaminobenzidine (DAKO) and counterstained with Gill I hematoxylin. Coverslipped slides were scanned with an Aperio ScanScope AT2 slide scanner (Leica Biosystems, Nussloch, Germany) using a 20× objective. For each marker, stained TMA sections were reviewed by breast pathologists to verify expected patterns of nuclear (ER, PR, Ki67, p16), membranous (HER2, CD20, CD3, CD45), or cytoplasmic (BCL2) staining in benign TDLUs and in BC cores before quantitative scoring [46,47]. The same TMAs were used for all cross-platform comparisons with DSP, summarized in Table 1.

### 2.5. Multiplex Immunofluorescence (OPAL)

OPAL staining (Akoya Biosciences, Marlborough, MA, USA) was performed on 4 µm sections using iterative antigen retrieval and tyramide signal amplification [48]. Prior to multiplexing, each primary antibody was validated on serial sections by conventional chromogenic immunohistochemistry and by singleplex immunofluorescence to confirm both staining pattern and optimal working dilution, first in reference tissues with known abundance of the target antigen and then in normal breast tissue [49,50,51]. Optimized conditions were carried forward into a six-color Opal protocol (Akoya Biosciences). Briefly, slides underwent sequential rounds of antigen retrieval, primary antibody incubation, HRP-conjugated secondary amplification and Opal fluorophore deposition, with microwave stripping (AR6 or 9 buffer, Akoya Biosciences, Marlborough, Massachusetts, USA) between cycles. A test slide containing all six antibodies was used to fine-tune marker order and Opal–antibody pairing, and to serve as a positive control in each staining batch. An autofluorescence-only slide received the full HRP cycle without Opal fluorophore and was included in spectral unmixing to characterize and isolate tissue autofluorescence.

The final panel was applied in the following sequence: CD4 (Abcam ab133616, 1:1000, Opal 480), Ki67 (Dako M7240, 1:100, Opal 520), CD8 (Sigma HPA037756, 1:2000, Opal 570), CD20 (Sigma HPA014341 1:3000, Opal 620), cytokeratin AE1/AE3 (Dako M3515, 1:150, Opal 690) and CD68 (HPA048982, 1:500, Opal 780). DAPI counterstain was applied in the final step. Slides were imaged on a PhenoImager HT 2.0 platform (Akoya Biosciences) using the manufacturer’s recommended exposure times, spectral libraries and autofocus settings. Multispectral images were unmixed with inForm 2.6; the autofluorescence library generated from the control slide was applied to all samples. Epithelial regions were delineated by cytokeratin signal, nuclei were segmented by DAPI and fine-tuned with assisting components, and marker-positive cells were identified with phenotype thresholds derived from single plex controls.

### 2.6. Statistical Methods

Associations of case-control status with demographic and clinical characteristics in the full BBD case-control set were assessed in the full BBD set using chi-square tests for categorical variables and two-sample t-tests for continuous variables.

**Figure 1:**
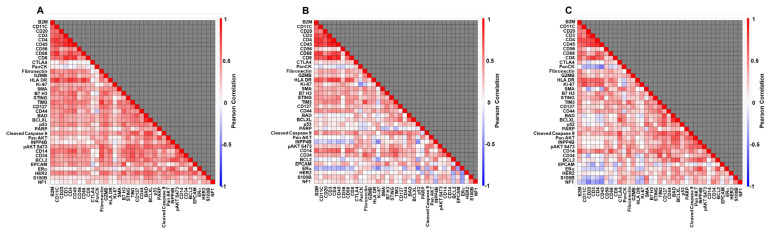
Marker–marker correlation matrices by tissue context. Correlograms display Pearson correlation coefficients among DSP protein biomarkers that passed quality control, computed per TMA core and shown separately for (**A**) benign breast TDLUs from the BBD case–control set, (**B**) breast cancers from women who later developed BC, and (**C**) TDLUs adjacent to those cancers. Each core was profiled with a single 600-µm circular ROI; expression values were log2-transformed before analysis. Warmer colors indicate stronger positive correlations (r → +1), cooler colors indicate negative correlations (r → –1). Expected immune relationships are evident across tissues (e.g., CD45 with T-cell, B-cell, macrophage, and dendritic-cell markers; HLA-DR with antigen-presenting cell markers), with comparatively weaker NK-cell (CD56) correlations in cancers. Detailed methods for ROI placement, antibody panels, QC, and correlation estimation are provided in the text.

Protein expression variables of interest included all original biomarkers provided by the DSP platform (see Figure 1). All expression values were transformed to the log(2) scale for all analyses. We filtered out low-expressing biomarkers by comparing expression to the three negative control biomarkers, mouse IgG1, mouse IgG2a and Rabbit IgG. For each biomarker, we calculated the mean difference between the log-transformed expression value and the average of the log-transformed negative control expression values across all TMA cores, and the proportion of times across all cores that the expression value was higher than all three positive control values. Biomarkers with mean differences less than zero and with expression values higher than the positive controls less than 5% of the time were excluded from all analyses.

For each biomarker that passed QC, we assessed reproducibility of log-expression across the multiple cores within a patient using intraclass correlation coefficients (ICCs) and corresponding 95% confidence intervals (CIs). ICCs were calculated within the three different tissue types (BBD-TDLUs, BC, and BC-associated TDLUs). We assessed per-core pairwise associations of biomarker log-expression values using Pearson correlation coefficients, overall and within the three different tumor types. Within the BBD TDLUs and BC lesions, we compared per-TMA core DSP and paired IHC expression (percent cells stained for CD20, CD3 and CD45 and H-score for BCL-2) and OPAL cell densities (stained cells per unit area for CD4, CD68, CD8 and Ki67). Data were categorized into quartiles (Q1; Q2 or 3; Q4) and assessed with weighted kappa statistics and 95% confidence intervals stratified by tissue type (BBD TDLUs, BC-associated TDLUs, BC). Agreement was categorized as 0.0–0.20 (slight); 0.21–0.40 (fair); 0.41–0.60 (moderate); 0.61–0.80 (substantial); 0.81–1.00 (nearly perfect) [52].

**Figure 2:**
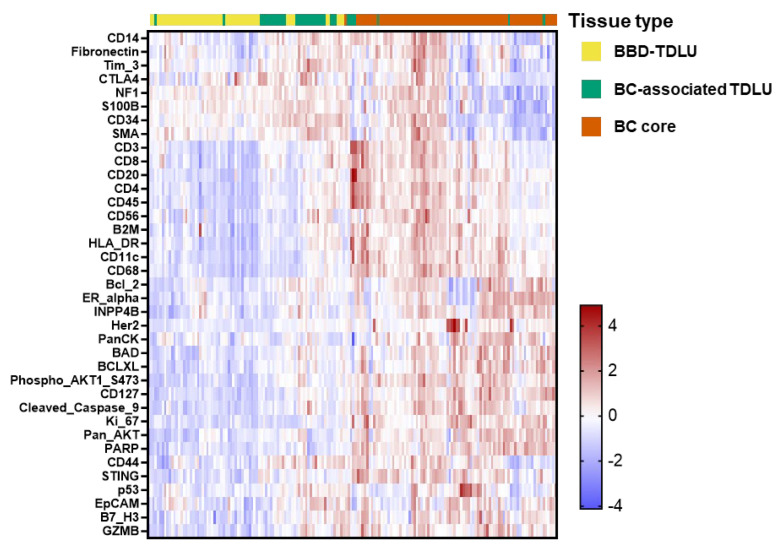
Tissue type gradients of DSP biomarkers among breast cancer cases. Heatmap summarizing standardized log2 protein expression across three tissue contexts sampled from women who developed breast cancer: benign TDLUs in the antecedent BBD biopsy (BBD TDLUs), morphologically benign lobules adjacent to the cancer at surgery (BC-associated TDLUs), and the cancer itself (BC cores). Each column represents a single TMA core (ROI) and each row represents one DSP biomarker (37 proteins that passed QC). Log2 expression values were standardized per biomarker (z score) across all case samples, such that values of 0 represent the mean expression for that marker, warmer colors indicate higher expression, and cooler colors indicate lower expression relative to the mean. Biomarkers and samples were ordered by unsupervised hierarchical clustering (Euclidean distance, Ward’s linkage) applied to the z-scored data. The colored bar above the heatmap indicates tissue type (yellow = BBD TDLUs, teal = BC-associated TDLUs, vermillion = BC cores). Many markers show gradients consistent with underlying biology (e.g., proliferation marker Ki67 is highest in cancers; several immune markers are highest in tumor-adjacent TDLUs). Log2 expression values were standardized per biomarker (z-score) across all case samples, such that values of 0 represent the mean expression for that marker, warmer colors indicate higher expression, and cooler colors indicate lower expression relative to the mean. The color bar, therefore, reflects the number of standard deviations above or below the biomarker-specific mean (standardized log2 expression).

We examined associations between expression values and the three tissue types in BBD cases using linear mixed models. Separate analyses were carried out for each biomarker, modeling log-expression values as the outcome variable and tissue type as the exposure. We accounted for the correlation of expression values across multiple cores within patients by fitting a per-patient random intercept term with a compound-symmetry (exchangeable) variance–covariance matrix. The resulting least-squares means and 95% confidence intervals were extracted for each of the three tissue types. Expression values by tissue type were visually compared using heatmaps. All analyses were conducted on background-adjusted, log2-transformed DSP expression values as described above. For heat-map visualization (Figure 2), these log2 values were further standardized per biomarker to z-scores across all case cores to emphasize relative up- and down-regulation rather than absolute count differences. For heat map visualization, we summarized DSP expression across all samples obtained from women who developed breast cancer, including BBD TDLUs, BC-associated TDLUs, and BC cores. For each of the 37 proteins that passed QC, Log2 expression values were standardized across all case samples by subtracting the biomarker-specific mean and dividing by the biomarker-specific standard deviation (z score). Unsupervised hierarchical clustering was then applied to both biomarkers (rows) and samples (columns) using Euclidean distance and Ward’s minimum variance linkage (Ward.D2), implemented in R. Clustering was used only to determine the ordering of rows and columns; no cluster cut points were used for formal inference.

In exploratory analyses, we examined associations between biomarker log-expression values in BBD and case status in the full case-control set, again using linear mixed models. Analyses modeled log-expression as the outcome variable, case status as the exposure variable and potential confounders as covariates. We accounted for the correlation in expression values across multiple cores within patients as described above. Associations were visually compared using log-fold changes in expression in cases vs. controls and corresponding 95% confidence intervals.

## 3. Results

### 3.1. Participant Characteristics

We analyzed 176 women: 88 BBD patients who later developed BC (“cases”) and 88 frequency-matched patients who did not develop BC (“controls”). From BBD biopsies, 368 TMA cores containing TDLUs were analyzed. Among the 88 cases, 52 had tissue from their subsequent BC surgeries, yielding a total of 204 BC cores and 110 cores from BC-associated TDLUs (Figure S1). Positive family history was more frequent among BBD-cases than controls (*p* = 0.003, Table S1). The remainder of self-reported characteristics and visually assessed TLDU involution did not vary by case-control status.

### 3.2. Digital Spatial Profiling (DSP) Quality Control and Reproducibility of DSP Measurements (ICCs)

Of the 79 assayed biomarkers, 40 (including housekeeping) passed QC based on comparison to isotype negative controls and were retained for analysis (most failures were low values). To assess the potential of DSP to detect marker heterogeneity among participants, we estimated ICCs for the three tissue types that were obtained from BC patients (TDLUs in BBD cases, BC, and BC-associated TDLUs; Table S2). ICCs were highest for BC tissues (range:0.30–0.92) and lower in BC-associated TDLUs (range: 0.06–0.51) and TDLUs in BBD biopsies (0.20–0.58). In BC tissues, 17 (45%) of 39 markers demonstrate ICCS ≥ 0.6. Clinical biomarkers in BC cores showed particularly strong reproducibility; specifically, for ER, ICC = 0.88 (95%CI: 0.81–0.92); for HER2, ICC = 0.92 (95%CI: 0.88–0.95) and for Ki67, ICC = 0.63 (95%CI: 0.49–0.75). In contrast, no marker exceeded ICC > 0.60 in TDLUs.

### 3.3. Biological Coherence: Correlation Among DSP Markers

Pairwise correlations (overall and by tissue type) demonstrated expected relationships among immunologic markers (Figure 1A–C). The pan-leukocyte marker CD45 correlated with lymphocyte subset markers, especially T lymphocytes (CD3, CD4, CD8), B lymphocytes (CD20), dendritic cells (CD11c), and macrophages (CD68). Similarly, the MHC class II molecule HLA-DR was correlated with antigen-presenting cell populations (substantially for CD11c, CD68, moderately for CD20) across all three tissue types, as expected. In BC tissues (Figure 1B), the natural killer cell marker CD56 showed weaker correlation with other markers than in TDLUs.

### 3.4. Cross-Platform Agreement: DSP Versus IHC and OPAL

We compared DSP with chromogenic IHC (ER, PR, BCL2, CD3, CD20, CD45) and OPAL immunofluorescence (CD4, CD8, CD68, Ki67) performed on the same TMAs using standard clinical antibodies and scoring criteria, and summarized the results using weighted kappa within each tissue context (Table 1). The IHC and OPAL stains showed the expected nuclear, membranous, or cytoplasmic localization patterns in benign TDLUs, BC-associated TDLUs, and BC cores, providing a familiar visual reference framework for interpreting the DSP measurements. Agreement in TDLUs was generally slight-to-fair, with ER and PR reaching fair agreement by IHC. Agreement in BC cores was high-to-moderate for ER and PR and substantial for BCL2. In BC-associated TDLUs, agreement exceeded that in BBD TDLUs and BC for some markers (CD3, CD45), reaching moderate concordance for CD45.

### 3.5. Spatial Tissue Type Contrasts Among Cases: BBD TDLUs, BC-Associated TDLUs, and BCs

Among women who developed BC, mixed effects models (per patient random intercept, compound symmetry) revealed multiple expression patterns across the three spatially distinct microenvironments: BBD TDLUs, BC-associated TDLUs, and BC cores (Table S3 and Figure S2). The most common pattern was higher expression in BC and in BC-associated TDLUs relative to TDLUs in the preceding BBD of the same patient, with representative *p*-values ranging from 2 × 10^−7^ for Bcl2 to 4 × 10^−45^ for CD68. Four markers exhibited higher levels in BC than in BC-associated TDLUs and lowest levels in TDLUs, including Ki67 (*p* = 3 × 10^−54^). T-cell and NK markers tended to be highest in BC-associated TDLUs, whereas the immune checkpoint proteins CTLA4 and Tim3 and the tumor suppressor NF1 were lowest in BC. Hierarchical clustering and heatmap visualization suggested three sub-clusters within BBD-TDLU cores, three within BC-associated TDLUs, and 4 clusters within BC cores, with BBD-TDLUs and BC-associated TDLUs clustering more closely to each other than to BC (Figure 2).

### 3.6. Exploratory Case–Control Analyses in BBD-TDLUs

In the full matched set (88 cases; 88 controls), BBD-TDLU expression of several markers was nominally higher in controls after adjustment for family history, including STING (*p* = 0.005), BCL2 (*p* = 0.008), CD44 (*p* = 0.02), and S100B (*p* = 0.05). None remained significant after controlling for false discovery rate (Benjamini–Hochberg). These analyses were not powered for risk prediction and are intended to inform assay and sampling choices for larger studies.

## 4. Discussion

Identifying biomarkers in BBD biopsies that predict BC risk could guide risk reduction strategies and identify pathways for targeted prevention [53,54,55,56], while providing reassurance for low-risk women. However, analyzing multiple biomarkers in small, formalin-fixed tissue structures such as TDLUs is inherently challenging, especially in percutaneous biopsies, which are sectioned at multiple levels for diagnosis, leaving minimal residual tissue for biomarker studies. To address this, we compared DSP-based protein profiling of TDLUs and BCs on TMAs with chromogenic IHC and OPAL immunofluorescence in a matched case-control subset from the Mayo Clinic BBD cohort. In TDLUs, agreement between DSP and other methods ranged from slight to fair, whereas agreement was considerably higher in BC cores. We also observed pronounced tissue type differences by DSP among cases, including BBD-TDLUs, BC-associated TDLUs, and BC. However, exploratory case–control contrasts in BBD-TDLUs did not persist after FDR control, indicating that, given current sampling and panel content, a univariable assessment of DSP protein measurements alone is unlikely to deliver near-term risk prediction.

Reproducibility patterns mirrored biological abundance and tissue context. ICCs were highest in BC cores, where canonical clinical markers such as ER and HER2 approached 0.9 and Ki-67 approached 0.6, and lower in BC-associated TDLUs and BBD-TDLUs. In benign lobules, lower target abundance and microanatomic heterogeneity likely attenuated reproducibility estimates. The observation that many TDLUs were highly involuted further supports this interpretation, as involution reduces glandular content and increases stromal replacement [12,13]. Together, these findings are consistent with DSP performing best where targets are abundant and spatially continuous (i.e., BC), while emphasizing the need for novel strategies to study benign structures.

Cross-platform agreement varied by tissue and marker class. In BC cores, where biomarker expression is often extensive and sampling differences between sections are less critical, we saw moderate (ER/PR) to substantial (BCL2) concordance. In TDLUs, agreement was generally slight to fair. Several methodological factors likely contributed: (i) DSP quantified a single 600 µm circular ROI per core (whole-ROI, no segmentation), whereas IHC/OPAL results effectively sampled the entire core area; (ii) section-to-section variation can shift sparse immune-cell signals across ordinal cut-points; and (iii) the inherent differences between assays: release/quantification of oligo tags (DSP) versus percent-positive cells (IHC) or cell densities (OPAL), limit direct one-to-one comparisons. While immunofluorescence typically offers higher dynamic range than chromogenic IHC, translating research-grade sensitivity to routine clinical practice remains non-trivial [29].

Given these constraints, it was striking that some biological structure in the DSP data remained coherent. Correlation matrices recapitulated known immunologic relationships: CD45 tracked with T-cell markers (CD3, CD4, CD8), B-cell marker CD20, with macrophage marker CD68, and a dendritic-cell marker (CD11c), while HLA-DR was associated with markers of antigen-presenting cells. This raises the idea that where individual markers may vary, multi-marker studies may circumvent some spatial variability and allow reliable detection of underlying biological processes [57,58,59,60,61]. In support of this view, tissue-type contrasts followed plausible gradients: many markers were highest in BC, intermediate in BC-associated TDLUs, and lowest in BBD-TDLUs; proliferative activity (Ki-67) showed a particularly strong BC-dominant gradient. Several observations were concordant with prior literature, including higher ER levels in BC than in BC-associated TDLUs or BBD-TDLUs and relatively increased ER in BC-associated TDLUs versus BBD-TDLUs [15]. We also noted lower CD20 levels in BBD-TDLUs that preceded BC compared with those in BC-associated TDLUs or BCs, aligning with our prior report linking a relative deficit of B cells in TDLUs of BBD biopsies with increased BC risk [21]. Enrichment of CD45, T cell markers, and the natural killer cell marker CD56 in BC-associated TDLUs relative to BCs or preceding BBD-TDLUs is of interest and merits further study [62,63]; related observation include reduced CD56-positive cells and decreased expression of its activating ligand MICA in BBD versus normal lobules [64], and associations between mutational burden and CD45+ cells in benign tissue [14].

These tissue type gradients also provide practical guidance for biomarker development and sampling strategies. First, markers whose expression increases monotonically from BBD TDLUs to BC-associated TDLUs to BC may report on progressive biologic changes along the benign–tumor continuum and could be prioritized as candidates for composite risk scores that integrate information across contexts. Second, the observation that many immune markers, including T cell and NK cell markers, are highest in BC-associated TDLUs suggests that tumor-adjacent TDLUs may be particularly informative for capturing host responses to nascent malignancy and might be preferentially sampled when tissue is limited [61,65,66,67,68]. Finally, the relative similarity between BBD TDLUs and BC-associated TDLUs compared to BC in the clustering analyses (Figure 2) indicates that benign lobules remain a distinct microanatomical compartment even in cancer-bearing breasts, reinforcing the value of TDLU-focused profiling for risk stratification while highlighting the importance of context-specific interpretation.

In exploratory case-control analyses restricted to BBD-TDLUs, several markers were nominally higher in controls (STING, BCL2, CD44 and S100B), but none remained significant after adjustment for multiple comparisons. These results, together with the ICC and agreement patterns above, suggest that risk-discriminating signals in benign lobules may be subtle relative to their biological and technical variability when assayed with a single, unsegmented ROI and a mid-sized protein panel. This emphasizes the importance of future studies that consider refined ROI strategies, including multiple or smaller epithelial-enriched ROIs and epithelial/stromal segmentation; expanded panels that also include RNA markers; and analytic frameworks that can leverage correlated features while remaining compatible with limited tissue and cohort-scale throughput.

Implementation of similar DSP on TMA strategies in larger and multi-institutional cohorts will require attention to several practical issues. Pre-analytic variability in fixation, processing, and block age can influence protein preservation and will need to be monitored and, where possible, harmonized across centers. Standardized protocols for TDLU-rich region selection, ROI placement, and quality control will be essential to reduce inter-observer and inter-site variability, particularly for benign lobules with variable involution [69,70]. Batch effects related to staining, instrument performance, and panel updates may become more prominent in larger studies and will require formal control designs and analytic adjustment. Finally, data sharing and analysis pipelines that can accommodate high plex, spatially indexed data at scale, while respecting patient privacy and institutional policies, will be important to enable multi-site discovery and validation efforts.

In summary, DSP on TMAs is technically feasible in this setting and yields biologically coherent profiles, with particularly robust performance in BC tissues. For benign lobules, low abundance and spatial heterogeneity constrain reproducibility and cross-platform agreement, and single-marker (univariable) DSP analyses alone did not yield FDR-robust case–control differences in this pilot. Expanding assay depth to include RNA and larger protein panels, integrating multiplex measurements across platforms, implementing technologies to assess biomarkers in single cells with spatial information, and expanding machine learning tools to provide meaning to spatial context are practical next steps that can be pursued without exhausting scarce tissue and that may ultimately support reproducible, clinically useful biomarkers for BBD risk stratification.

## 5. Conclusions

TMA-based DSP is feasible in archival breast tissues and produces biologically coherent profiles across benign lobules, tumor-adjacent lobules, and cancers, with particularly robust performance in cancers and solid cross-platform benchmarks against IHC/OPAL. In benign lobules, subtle signals amid microanatomic heterogeneity and lower target abundance constrained single-marker reproducibility and case–control discrimination in this pilot, emphasizing the need for refined ROI strategies, segmentation, and expanded panels (including RNA) in future studies. These results provide a practical blueprint for scaling TDLU-focused spatial profiling, via optimized sampling and multi-analyte analytics, to accelerate discovery of reproducible, clinically useful biomarkers for risk stratification after BBD while conserving scarce tissue resources.

## Figures and Tables

**Table 1:** Weighted kappa statistics and percent exact agreement between DSP expression and paired chromogenic IHC or OPAL immunofluorescence for selected biomarkers on TMA cores. IHC measures are percent of cells positive (ER, PR, CD3, CD20, CD45) or H score (BCL2); OPAL measures are cell densities (cells per unit area) for CD4, CD8, CD68, and Ki67. These values provide representative samples of IHC and OPAL results on the TMAs across the three tissue contexts (BBD TDLUs, BC-associated TDLUs, BC cores).

		Weighted Kappa (95% CI) Exact Agreement		
Panel	Biomarker	BBD TDLUs	BC	BC-Associated TDLUs
IHC	BCL-2 H score	0.05 (−0.05–0.14) 44.7%	0.61 (0.49–0.72) 67.8%	0.15 (−0.07–0.37) 47.7%
IHC	CD20 percent cells positive	0.09 (−0.02–019) 41.4%	0.22 (0.09–0.34) 53.6%	N/A 48.7%
IHC	CD3 percent cells positive	0.17 (0.07–0.27) 47.1%	0.20 (0.06–0.34) 44.1%	0.26 (0.28–0.45) 55.1%
IHC	CD45 percent cells positive	0.16 (0.05–0.27) 48.5%	0.27 (0.14–0.40) 47.8%	0.43 (0.05–0.48) 55.1%
IHC	ER percent cells positive	0.32 (0.21–0.43) 61.8%	0.73 (0.62–0.84) 80.4%	0.26 (0.04–0.49) 53.1%
IHC	PR percent cells positive	0.24 (0.14–0.35) 54.1%	0.53 (0.42–0.65) 60.8%	0.26 (0.06–0.46) 48.2%
OPAL	CD4 cell density	0.0 (−0.09–0.09) 41.7%	0.14 (−0.00–0.28) 42.3%	N/A 46.9%
OPAL	CD68 cell density	0.13 (0.04–0.22) 50.3%	−0.02 (−0.19–0.15) 48.6%	N/A 51.6%
OPAL	CD8 cell density	0.07 (−0.01–0.16) 42.7%	0.10 (−0.03–0.22) 44.1%	0.10 (−0.06–0.27) 42.2%
OPAL	Ki67 cell density	0.10 (0.01–0.20) 47.7%	N/A 68.4%	N/A 46.9%

## Data Availability

All data will be made available upon reasonable request.

## Supplementary Materials

The following supporting information can be downloaded at: https://www.mdpi.com/article/10.3390/cancers17233797/s1, Figure S1. Study cohort and assay flow (CONSORT-style diagrams). Panel A shows sample selection and numbers for the full case–control set and the case-only set; Panel B shows biomarker QC filtering (79 assayed; 40 passed QC, including housekeeping; 37 non-control proteins retained after removing positive controls). Figure S2. Associations between biomarker expression and tissue type among breast cancer cases. Linear mixed models with per-patient random intercepts; results visualized across benign, tumor-adjacent, and tumor tissues. Table S1. Associations of breast cancer case status with demographic and clinical characteristics in the full benign case–control set. Table S2. Intraclass correlation coefficients of log-transformed DSP biomarker expression across TMA cores per patient, by tissue type and overall. Table S3. Among breast cancer cases, the least-squares means of DSP biomarkers by tissue type (benign lobules, tumor-adjacent lobules, cancers), with 95% CIs and *p*-values.

## Abbreviations

The following abbreviations are used in this manuscript:

AH	Atypical hyperplasia
BC	Breast cancer
BBD	Benign breast disease
BCL2/BCL-XL	B-cell lymphoma 2/B-cell lymphoma extra-long isoform
CI	Confidence interval
DAB	3,3′-diaminobenzidin
DAPI	4′,6-diamidino-2-phenylindole
DSP	Digital Spatial Profiling
ER/PR/HER2	Estrogen receptor/Progesterone receptor/Human epidermal growth factor receptor 2
FDR	False discovery rate
FFPE	Formalin-fixed paraffin-embedded
H&E	Hematoxylin and eosin
H-score	Histologic score (semi-quantitative immunostain metric)
HRP	Horseradish peroxidase
ICC	Intraclass correlation coefficient
IHC	Immunohistochemistry (chromogenic)
IGF1R	Insulin-like growth factor 1 receptor
LS-means	Least-squares means
MHC	Major histocompatibility complex
NK	Natural killer (cell)
OPAL	Tyramide-based multiplex immunofluorescence platform
QC	Quality control
ROI	Region of interest
SMA	Smooth muscle actin
STING	Stimulator of interferon genes
TDLU	Terminal duct lobular unit
TMA	Tissue microarray
