# Supplementary material for: Tissue Microarray-Based Digital Spatial Profiling of Benign Breast Lobules and Breast Cancers: Feasibility, Biological Coherence, and Cross-Platform Benchmarks
Source: Cancers (Basel). 2025 Nov 27;17(23):3797. doi: 10.3390/cancers17233797 (PMC12691068; doi:10.3390/cancers17233797)

Supplemental Table S1. Associations of breast cancer case status with demographic and clinical characteristics.

	Breast cancer case status			
	Case (N=88)	Control (N=88)	Total (N=176)	P-value
**Age at initial benign biopsy**				0.639[1]
N	88	88	176	
Mean (SD)	51.7 (8.79)	52.3 (10.07)	52.0 (9.43)	
Median	50.0	51.0	51.0	
Range	36.0, 71.0	19.0, 77.0	19.0, 77.0	
**Body mass index**				0.210[1]
N	88	88	176	
Mean (SD)	26.9 (6.51)	28.2 (6.89)	27.6 (6.72)	
Median	26.0	27.0	26.0	
Range	18.0, 60.0	18.0, 58.0	18.0, 60.0	
**Histologic impression**, n (%)				0.646[2]
AH	24 (27.3%)	25 (28.4%)	49 (27.8%)	
NP	21 (23.9%)	16 (18.2%)	37 (21.0%)	
PDWA	43 (48.9%)	47 (53.4%)	90 (51.1%)	
**Extent of lobular involution**, n (%)				0.244[2]
< 25%	29 (33.3%)	22 (25.3%)	51 (29.3%)	
>= 75%	58 (66.7%)	65 (74.7%)	123 (70.7%)	
Missing	1	1	2	
**Family history of breast cancer**, n (%)				<.001[2]
Negative	27 (31.4%)	48 (57.1%)	75 (44.1%)	
Positive	59 (68.6%)	36 (42.9%)	95 (55.9%)	
Missing	2	4	6	
**Parity**, n (%)				0.882[2]
Nulliparous	11 (12.6%)	11 (13.4%)	22 (13.0%)	
Parous	76 (87.4%)	71 (86.6%)	147 (87.0%)	
Missing	1	6	7	
**Parity/age at first birth**, n (%)				0.649[2]
Nulliparous	11 (12.8%)	11 (13.8%)	22 (13.3%)	
1-2, < 30 at first	31 (36.0%)	28 (35.0%)	59 (35.5%)	
1-2, >= 30 at first	9 (10.5%)	6 (7.5%)	15 (9.0%)	
3+, M 30 at first	31 (36.0%)	34 (42.5%)	65 (39.2%)	
3+, >= 30 at first	4 (4.7%)	1 (1.3%)	5 (3.0%)	
Missing	2	8	10	
**Parity/breastfeeding**, n (%)				0.117[2]
Nulliparous	11 (14.3%)	11 (14.5%)	22 (14.4%)	
Parous, no breastfeeding	34 (44.2%)	45 (59.2%)	79 (51.6%)	
Parous, breastfeeding	32 (41.6%)	20 (26.3%)	52 (34.0%)	
Missing	11	12	23	
**Menopausal status**, n (%)				0.642[2]
Peri-Menopausal	1 (1.1%)	2 (2.3%)	3 (1.7%)	
Post-Menopausal	84 (95.5%)	81 (92.0%)	165 (93.8%)	
Pre-Menopausal	3 (3.4%)	5 (5.7%)	8 (4.5%)	
**Use of hormone replacements**, n (%)				0.349[2]
Current	4 (4.6%)	9 (11.1%)	13 (7.7%)	

	Breast cancer case status			
	Case (N=88)	Control (N=88)	Total (N=176)	P-value
Ever, unsure if current	4 (4.6%)	3 (3.7%)	7 (4.2%)	
Former	46 (52.9%)	45 (55.6%)	91 (54.2%)	
Never	33 (37.9%)	24 (29.6%)	57 (33.9%)	
Missing	1	7	8	

[1]Equal variance two sample t-test; [2]Chi-Square p-value;

Supplemental Table S2. Intraclass correlation coefficients (ICCs) of log-transformed DSP biomarker expression across the multiple TMA cores per patient, overall and by tissue type.

Biomarker	BBD TDLUs ICC (95% CI)	BC ICC (95% CI)	BC-associated TDLUs ICC (95% CI)	All ICC (95% CI)
B2M	0.28 (0.18,0.37)	0.59 (0.43,0.72)	0.08 (-.05,0.26)	0.30 (0.25,0.36)
CD11c	0.26 (0.16,0.35)	0.62 (0.47,0.74)	0.21 (0.06,0.40)	0.38 (0.33,0.44)
CD20	0.27 (0.18,0.37)	0.30 (0.13,0.48)	0.25 (0.09,0.44)	0.21 (0.16,0.26)
CD3	0.21 (0.11,0.31)	0.44 (0.27,0.61)	0.29 (0.13,0.49)	0.14 (0.10,0.19)
CD4	0.32 (0.23,0.42)	0.55 (0.39,0.70)	0.30 (0.14,0.50)	0.23 (0.18,0.29)
CD45	0.33 (0.24,0.43)	0.55 (0.39,0.69)	0.39 (0.22,0.57)	0.23 (0.18,0.28)
CD56	0.27 (0.18,0.37)	0.41 (0.24,0.58)	0.17 (0.02,0.36)	0.21 (0.16,0.26)
CD68	0.20 (0.11,0.30)	0.58 (0.42,0.71)	0.15 (0.00,0.34)	0.32 (0.26,0.38)
CD8	0.30 (0.20,0.39)	0.55 (0.39,0.69)	0.22 (0.07,0.41)	0.20 (0.15,0.25)
CTLA4	0.26 (0.17,0.36)	0.51 (0.34,0.66)	0.28 (0.12,0.48)	0.21 (0.16,0.26)
PanCk	0.33 (0.24,0.43)	0.66 (0.52,0.78)	0.51 (0.34,0.67)	0.27 (0.22,0.33)
Fibronectin	0.28 (0.19,0.38)	0.51 (0.34,0.66)	0.06 (-.06,0.24)	0.11 (0.07,0.16)
GZMB	0.34 (0.25,0.44)	0.42 (0.25,0.59)	0.17 (0.02,0.36)	0.24 (0.19,0.29)
HLA DR	0.30 (0.21,0.40)	0.59 (0.44,0.73)	0.34 (0.17,0.53)	0.30 (0.25,0.36)
Ki 67	0.27 (0.18,0.37)	0.63 (0.49,0.75)	0.33 (0.17,0.52)	0.46 (0.40,0.52)
SMA	0.27 (0.18,0.37)	0.57 (0.41,0.71)	0.30 (0.14,0.50)	0.17 (0.12,0.22)
B7H3	0.55 (0.47,0.63)	0.61 (0.46,0.74)	0.42 (0.25,0.60)	0.37 (0.32,0.43)
STING	0.22 (0.12,0.32)	0.63 (0.48,0.75)	0.22 (0.07,0.42)	0.21 (0.16,0.27)
Tim3	0.22 (0.12,0.32)	0.60 (0.44,0.73)	0.19 (0.04,0.38)	0.16 (0.11,0.21)
CD127	0.26 (0.16,0.36)	0.58 (0.42,0.71)	0.16 (0.02,0.36)	0.33 (0.27,0.39)
CD44	0.58 (0.50,0.66)	0.61 (0.46,0.74)	0.44 (0.27,0.62)	0.32 (0.26,0.38)
BAD	0.22 (0.12,0.31)	0.71 (0.59,0.81)	0.25 (0.09,0.45)	0.23 (0.18,0.29)
BCLXL	0.25 (0.16,0.35)	0.55 (0.39,0.69)	0.35 (0.18,0.54)	0.24 (0.19,0.30)
p53	0.28 (0.19,0.38)	0.66 (0.52,0.78)	0.38 (0.21,0.57)	0.15 (0.11,0.20)
PARP	0.22 (0.13,0.32)	0.71 (0.58,0.81)	0.29 (0.12,0.48)	0.40 (0.34,0.46)
Cleaved_Caspase_9	0.26 (0.16,0.36)	0.54 (0.38,0.68)	0.36 (0.19,0.55)	0.26 (0.21,0.32)
Pan_AKT	0.27 (0.18,0.37)	0.78 (0.68,0.86)	0.40 (0.23,0.58)	0.31 (0.26,0.37)
INPP4B	0.30 (0.21,0.40)	0.58 (0.42,0.72)	0.24 (0.08,0.43)	0.27 (0.22,0.32)
Phospho_AKT1_S473	0.20 (0.10,0.30)	0.53 (0.37,0.68)	0.12 (-.01,0.31)	0.23 (0.18,0.29)
CD14	0.36 (0.27,0.46)	0.57 (0.41,0.71)	0.13 (-.01,0.32)	0.12 (0.08,0.17)
CD34	0.37 (0.27,0.46)	0.44 (0.27,0.61)	0.46 (0.29,0.64)	0.21 (0.17,0.27)
Bcl_2	0.38 (0.29,0.48)	0.74 (0.62,0.83)	0.24 (0.09,0.44)	0.29 (0.24,0.35)
EpCAM	0.47 (0.38,0.56)	0.64 (0.50,0.76)	0.19 (0.04,0.39)	0.25 (0.20,0.31)
ER_alpha	0.39 (0.29,0.48)	0.88 (0.81,0.92)	0.38 (0.21,0.56)	0.37 (0.32,0.43)
Her2	0.40 (0.31,0.49)	0.92 (0.88,0.95)	0.43 (0.26,0.61)	0.36 (0.31,0.42)

Biomarker	BBD TDLUs ICC (95% CI)	BC ICC (95% CI)	BC-associated TDLUs ICC (95% CI)	All ICC (95% CI)
S100B	0.39 (0.30,0.48)	0.58 (0.43,0.72)	0.45 (0.28,0.63)	0.23 (0.18,0.29)
NF1	0.39 (0.29,0.48)	0.57 (0.42,0.71)	0.41 (0.24,0.59)	0.16 (0.12,0.21)

Supplemental Table S3. Among BC cases, associations between DSP biomarker expression values and tissue type, sorted by expression patterns. Associations expressed as least squares means and 95% CIs, accounting for multiple cores within a subject and adjusted for family history of breast cancer.

Biomarker	Tissue type	Mean of log2 expression (95% CI)	p-value
**Expression in BC and BC-associated cores is higher, expression in BBD cores is lower**			
B2M	BC cores	8.38 (8.26 , 8.51)	7E-24
B2M	BC-associated cores	8.54 (8.37 , 8.70)	
B2M	BBD cores	7.63 (7.49 , 7.76)	
Bcl_2	BC cores	9.30 (9.08 , 9.51)	2E-7
Bcl_2	BC-associated cores	9.48 (9.21 , 9.74)	
Bcl_2	BBD cores	8.84 (8.61 , 9.07)	
CD11c	BC cores	8.85 (8.71 , 8.99)	4E-42
CD11c	BC-associated cores	8.80 (8.62 , 8.97)	
CD11c	BBD cores	7.64 (7.49 , 7.78)	
CD20	BC cores	8.30 (8.15 , 8.44)	2E-23
CD20	BC-associated cores	8.56 (8.37 , 8.75)	
CD20	BBD cores	7.40 (7.24 , 7.56)	
CD68	BC cores	9.56 (9.44 , 9.69)	4E-45
CD68	BC-associated cores	9.75 (9.58 , 9.91)	
CD68	BBD cores	8.39 (8.25 , 8.52)	
Cleaved_Caspase_9	BC cores	10.57 (10.46 , 10.69)	1E-23
Cleaved_Caspase_9	BC-associated cores	10.37 (10.23 , 10.52)	
Cleaved_Caspase_9	BBD cores	9.81 (9.69 , 9.94)	
ER_alpha	BC cores	9.06 (8.57 , 9.55)	8E-19
ER_alpha	BC-associated cores	8.37 (7.79 , 8.96)	
ER_alpha	BBD cores	6.79 (6.28 , 7.30)	
GZMB	BC cores	9.33 (9.19 , 9.47)	8E-17
GZMB	BC-associated cores	9.07 (8.89 , 9.24)	
GZMB	BBD cores	8.56 (8.41 , 8.70)	
HLA_DR	BC cores	9.43 (9.29 , 9.57)	1E-24
HLA_DR	BC-associated cores	9.57 (9.40 , 9.75)	
HLA_DR	BBD cores	8.64 (8.50 , 8.79)	
INPP4B	BC cores	8.69 (8.48 , 8.90)	2E-20
INPP4B	BC-associated cores	8.35 (8.08 , 8.61)	
INPP4B	BBD cores	7.43 (7.21 , 7.66)	
Phospho_AKT1_S473	BC cores	7.56 (7.42 , 7.70)	2E-25
Phospho_AKT1_S473	BC-associated cores	7.59 (7.41 , 7.78)	

Biomarker	Tissue type	Mean of log2 expression (95% CI)	p-value
Phospho_AKT1_S473	BBD cores	6.53 (6.38 , 6.68)	
STING	BC cores	8.25 (8.07 , 8.44)	6E-17
STING	BC-associated cores	8.59 (8.36 , 8.82)	
STING	BBD cores	7.51 (7.31 , 7.70)	
**Expression in BC cores is higher, expression in BC-associated cores is intermediate, expression in BBD cores is lower**			
CD127	BC cores	8.86 (8.72 , 9.00)	8E-51
CD127	BC-associated cores	8.20 (8.04 , 8.37)	
CD127	BBD cores	7.51 (7.37 , 7.66)	
Ki_67	BC cores	9.04 (8.87 , 9.22)	3E-54
Ki_67	BC-associated cores	8.18 (7.96 , 8.40)	
Ki_67	BBD cores	7.06 (6.88 , 7.25)	
PARP	BC cores	9.21 (9.05 , 9.37)	4E-48
PARP	BC-associated cores	8.60 (8.39 , 8.80)	
PARP	BBD cores	7.40 (7.23 , 7.58)	
Pan_AKT	BC cores	10.32 (10.17 , 10.47)	2E-28
Pan_AKT	BC-associated cores	9.72 (9.52 , 9.91)	
Pan_AKT	BBD cores	9.16 (8.99 , 9.32)	
**Expression in BC cores tissue is higher, expression in adjacent BC-associated cores tissue and benign tissue is lower**			
B7_H3	BC cores	9.78 (9.51 , 10.06)	2E-24
B7_H3	BC-associated cores	8.90 (8.57 , 9.22)	
B7_H3	BBD cores	8.41 (8.12 , 8.69)	
BAD	BC cores	10.29 (10.14 , 10.43)	3E-22
BAD	BC-associated cores	9.67 (9.48 , 9.85)	
BAD	BBD cores	9.41 (9.26 , 9.57)	
BC coresLXL	BC cores	9.56 (9.42 , 9.70)	5E-32
BC coresLXL	BC-associated cores	8.65 (8.47 , 8.83)	
BC coresLXL	BBD cores	8.41 (8.26 , 8.56)	
EpCAM	BC cores	8.76 (8.43 , 9.08)	4E-12
EpCAM	BC-associated cores	7.41 (7.01 , 7.81)	
EpCAM	BBD cores	7.74 (7.40 , 8.08)	
Her2	BC cores	8.38 (7.98 , 8.77)	7E-26
Her2	BC-associated cores	6.98 (6.53 , 7.44)	
Her2	BBD cores	6.38 (5.97 , 6.79)	
PanCk	BC cores	13.30 (13.07 , 13.53)	6E-20

Biomarker	Tissue type	Mean of log2 expression (95% CI)	p-value
PanCk	BC-associated cores	12.47 (12.18 , 12.75)	
PanCk	BBD cores	11.96 (11.72 , 12.20)	
**Expression in adjacent BC-associated cores tissue is higher, expression in BC cores tissue is intermediate, expression in benign tissue is lower**			
CD3	BC cores	8.08 (7.94 , 8.23)	2E-15
CD3	BC-associated cores	8.63 (8.43 , 8.82)	
CD3	BBD cores	7.64 (7.49 , 7.80)	
CD4	BC cores	8.81 (8.67 , 8.95)	7E-29
CD4	BC-associated cores	9.39 (9.21 , 9.57)	
CD4	BBD cores	8.16 (8.01 , 8.31)	
CD45	BC cores	9.59 (9.43 , 9.75)	7E-24
CD45	BC-associated cores	10.18 (9.97 , 10.39)	
CD45	BBD cores	8.83 (8.66 , 9.00)	
CD56	BC cores	8.46 (8.35 , 8.57)	5E-31
CD56	BC-associated cores	8.90 (8.76 , 9.04)	
CD56	BBD cores	7.80 (7.69 , 7.92)	
CD8	BC cores	8.83 (8.69 , 8.97)	7E-14
CD8	BC-associated cores	9.37 (9.19 , 9.56)	
CD8	BBD cores	8.48 (8.33 , 8.64)	
**Expression in adjacent BC-associated cores tissue is higher, expression in BC cores tissue and benign tissue is lower**			
Fibronectin	BC cores	12.14 (11.86 , 12.42)	1E-9
Fibronectin	BC-associated cores	13.44 (13.08 , 13.80)	
Fibronectin	BBD cores	12.35 (12.05 , 12.65)	
**Expression in adjacent BC-associated cores and benign tissue is higher, expression in BC cores tissue is lower**			
CD34	BC cores	8.14 (7.91 , 8.37)	2E-55
CD34	BC-associated cores	11.07 (10.77 , 11.37)	
CD34	BBD cores	10.72 (10.47 , 10.96)	
CTLA4	BC cores	7.10 (6.86 , 7.33)	6E-13
CTLA4	BC-associated cores	7.95 (7.65 , 8.26)	
CTLA4	BBD cores	8.19 (7.94 , 8.44)	
NF1	BC cores	7.09 (6.87 , 7.32)	7E-43
NF1	BC-associated cores	9.58 (9.29 , 9.88)	
NF1	BBD cores	9.19 (8.95 , 9.43)	
S100B	BC cores	7.80 (7.48 , 8.12)	1E-35

Biomarker	Tissue type	Mean of log2 expression (95% CI)	p-value
S100B	BC-associated cores	10.38 (9.98 , 10.78)	
S100B	BBD cores	10.15 (9.81 , 10.48)	
SMA	BC cores	13.47 (13.25 , 13.69)	1E-23
SMA	BC-associated cores	14.89 (14.60 , 15.17)	
SMA	BBD cores	14.69 (14.45 , 14.92)	
Tim_3	BC cores	7.41 (7.23 , 7.59)	2E-10
Tim_3	BC-associated cores	8.19 (7.95 , 8.42)	
Tim_3	BBD cores	8.04 (7.85 , 8.24)	
**Expression in adjacent BC-associated cores tissue is higher, expression in benign tissue is intermediate, expression in BC cores tissue is lower**			
CD14	BC cores	7.70 (7.50 , 7.90)	1E-17
CD14	BC-associated cores	9.07 (8.81 , 9.33)	
CD14	BBD cores	8.19 (7.97 , 8.40)	
CD44	BC cores	10.63 (10.39 , 10.88)	61E-6
CD44	BC-associated cores	11.41 (11.09 , 11.73)	
CD44	BBD cores	10.84 (10.58 , 11.11)	
**Expression in BC cores tissue is higher, expression in benign tissue is intermediate, expression in adjacent BC-associated cores tissue is lower**			
p53	BC cores	9.60 (9.37 , 9.83)	85E-5
p53	BC-associated cores	9.01 (8.71 , 9.30)	
p53	BBD cores	9.35 (9.10 , 9.59)	

Figure S1. Study CONSORT-style diagrams for cohort selection and biomarker QC. (A) Patients: Flow of samples contributing to the benign case–control analyses (368 ROIs from benign tissue; 176 unique participants—88 cases and 88 controls) and the case-only analyses (110 ROIs from benign tissue; 204 ROIs from tumor blocks—133 tumor and 71 adjacent normal—from 52 cases with available surgical material). **(B) Biomarkers:** Of 79 proteins assayed by DSP, 40 (including housekeeping/positive controls) passed QC; after removing positive control biomarkers (GAPDH, Histone H3, S6), 37 non-control proteins were retained for analysis. “Passed QC” reflects filtering by comparison to isotype negatives and proportion above positive controls, as detailed in Methods.

A

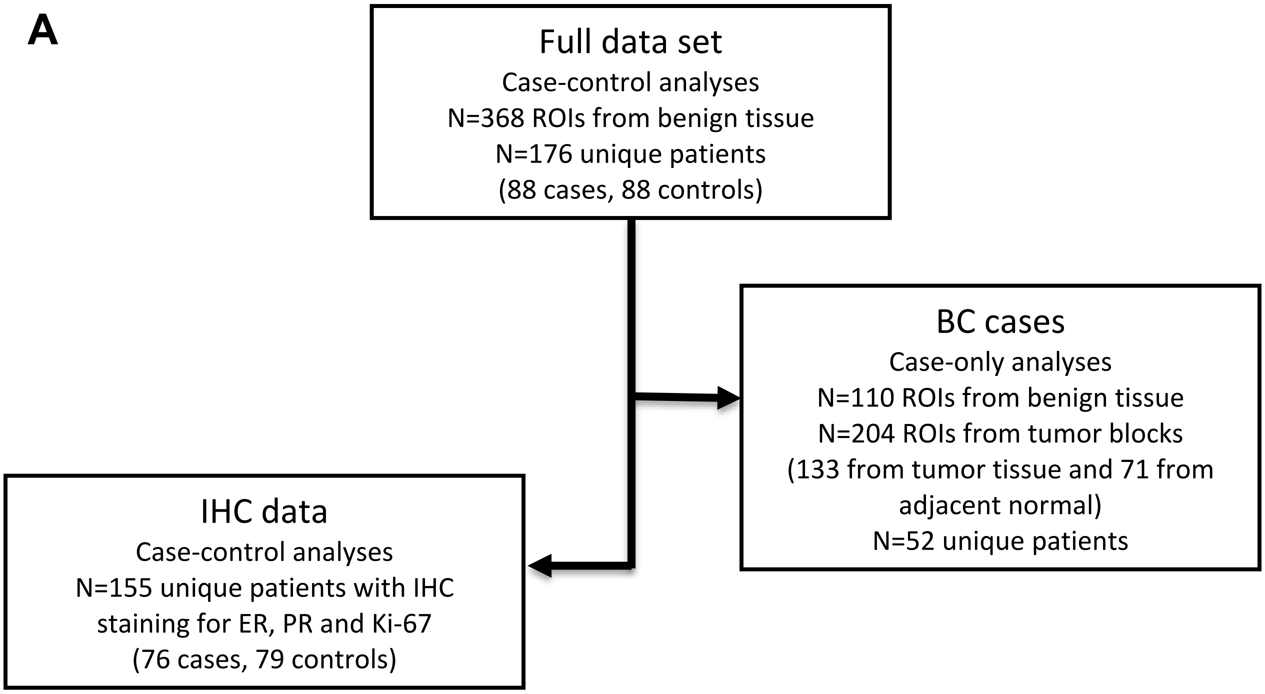

B

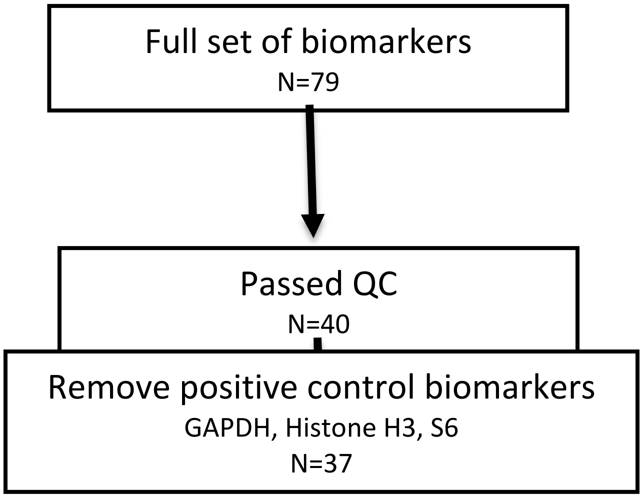

Figure S2. Linear mixed-model contrasts of biomarker expression by tissue type among cases. Per-marker comparisons of log2 DSP expression across benign TDLUs, tumor-adjacent TDLUs, and cancers in women who developed BC. Models include a random intercept for subject ID to account for multiple TMA cores per patient; least-squares means and 95% CIs are shown. Results illustrate patterns summarized in the main text and Figure 2 (e.g., cancer-dominant proliferation; immune-cell markers often enriched in tumor-adjacent TDLUs)

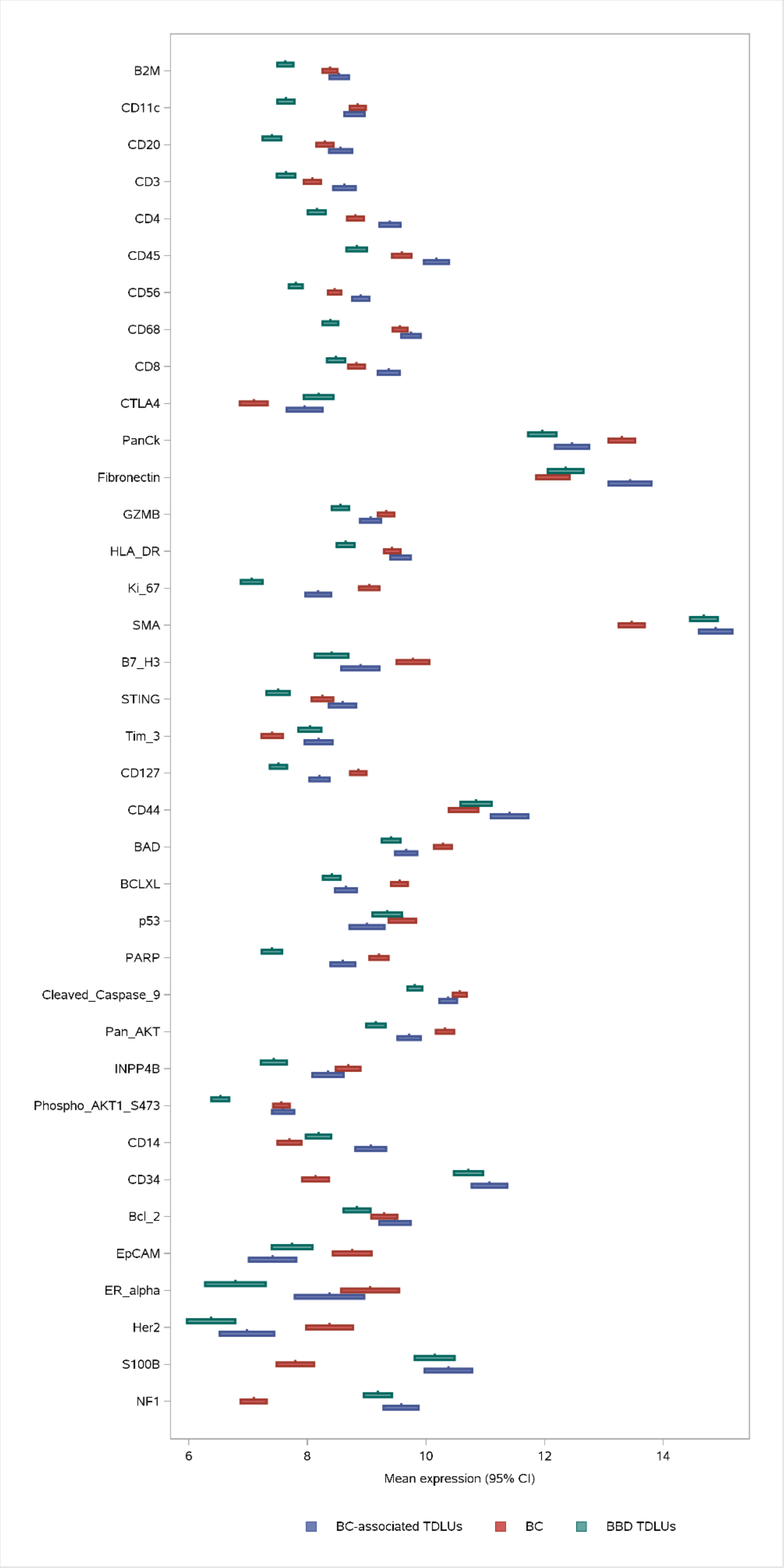

Supplement: Supplementary file 1 [file cancers-17-03797-s001.zip › cancers-3995629-supplementaryl.pdf]
